# Rice Bran-Derived Peptides with Antioxidant Activity: Effects of Enzymatic Hydrolysis Using *Bacillus licheniformis* and α-Chymotrypsin

**DOI:** 10.3390/foods15030516

**Published:** 2026-02-02

**Authors:** Rodjana Noptana, David Julian McClements, Lynne A. McLandsborough, Wiriya Onsaard, Ekasit Onsaard

**Affiliations:** 1 Faculty of Agriculture, Ubon Ratchathani University, Ubon Ratchathani 34190, Thailand; rodjana.no.62@ubu.ac.th (R.N.); wiriya.p@ubu.ac.th (W.O.); 2 Biopolymers & Colloids Research Laboratory, Department of Food Science, University of Massachusetts, Amherst, MA 01003, USA; mcclemen@umass.edu; 3 Food Microbiology, Department of Food Science, University of Massachusetts, Amherst, MA 01003, USA; lm@umass.edu; 4 Indigenous Food Research and Industrial Development Center, Ubon Ratchathani University, Ubon Ratchathani 34190, Thailand

**Keywords:** rice bran protein hydrolysate, protein fractions, bioactive compounds, antioxidant activity, circular economy

## Abstract

Rice bran, a nutrient-rich by-product of rice milling, is an underutilized resource in sustainable crop utilization. This study aimed to investigate the characteristics, total phenolic content, and antioxidant activities of rice bran protein hydrolysates (RBPHs) produced using proteases from *Bacillus licheniformis* (RBPH-B) and α-chymotrypsin (RBPH-C), along with their protein fractions (F1; >100 kDa, F2; 10–100 kDa, F3; 1–10 kDa, F4; <1 kDa). Molecular weight, color, surface hydrophobicity, secondary structure, total phenolic content, and antioxidant activities of the hydrolysates were assessed. Both enzymatic hydrolysis and ultrafiltration reduced molecular weight and surface hydrophobicity, enhanced lightness, and increased α-helix content. Among all samples, the <1 kDa peptide fraction derived from α-chymotrypsin hydrolysis (RBPH-C-F4) exhibited the strongest antioxidant activity, with the lowest EC_50_ values for ABTS (0.94 mg/mL) and DPPH (210 µg/mL), as well as the highest inhibition of metal chelating activity (1.35 mmol EDTA/g sample) and linoleic peroxidation (90.62%). Enzymatic hydrolysis enhanced total phenolic content compared with native rice bran protein. These findings highlight the potential of rice bran-derived peptides as antioxidant candidates and indicate that further validation in food systems is required.

## 1. Introduction

Rice milling generates large quantities of rice bran, a peripheral grain that is commonly underutilized despite its substantial nutrition potential [1]. Rice bran contains a rich source of nutrients, including protein (11–17%), oil (12–22%), fiber (6–14%), minerals (8–17%), and vitamins, including tocopherols, thiamine, and niacin [2]. Rice bran protein is characterized by relatively high proportion of essential amino acids, particularly aromatic amino acids (9.46–11.41%), which are wildly associated with antioxidant activity [1,3]. However, despite its nutritional value, the direct incorporation of rice bran into food products remains limited, primarily due to its high lipid content, which accelerates oxidative deterioration and reduces shelf stability. As a result, defatted rice bran has attracted as a protein-rich substrate suitable for further functional modification and value-added utilization.

Proteins derived from cereal by-products possess latent bioactivity that can be unlocked through enzymatic hydrolysis. During proteolysis, specific peptide sequences embedded within native protein structures are released, generating short peptide that may exhibit antioxidant, metal-chelating, or radical-scavenging functions [3,4]. Various enzymes, including subtilisin A from *Bacillus licheniformis* [5], papain [1], α-chymotrypsin, flavoenzyme [6], pepsin, and trypsin [3], have been employed to generate bioactive hydrolysates from rice bran [7]. Previous studies have demonstrated that antioxidant activitie is influenced by peptide molecular weight, amino acid composition, and hydrophobicity, with low molecular weight fractions often exhibiting enhanced activity due to improved electron donation, radical stabilization, and metal-chelation capacity. Despite these advances, several knowledge gaps remain. Most previous studies have focused on single enzyme or bulk hydrolysate, with limited direct comparison of protease with distinct cleavage specificities. Although peptide size fractionation has been reported, systematic structure-function analysis across defined molecular weight fractions generated by different enzymes remains scare. Importantly, the combined influence of enzyme specificity, peptide molecular size distribution, and antioxidant efficacy in rice bran protein systems remains insufficiently understood.

Therefore, the present study was designed to test the following hypotheses: (i) proteases with distinct cleavage specificities (*Bacillus licheniformis* versus α-chymotrypsin) generate peptide populations with different molecular weight distributions and physicochemical characteristics; (ii) peptide molecular weight fractionation significantly influences antioxidant mechanism, including radical scavenging, metal chelation, and lipid peroxidation inhibition; and (iii) the observed differences in antioxidant activity can be correlated with enzyme specificity-dependent changes in peptide size distribution and structural properties. In this work, we aim to provide mechanistic insight into the valorization of rice bran protein as functional ingredients, contributing to sustainable utilization of agriculture by-products and development of plant-derived antioxidant for food applications.

## 2. Materials and Methods

### 2.1. Materials

Fresh rice bran was obtained from a local rice milling facility in Det Udom district, Ubon Ratchathani province, Thailand. The proximate compositions of rice bran were reported on a dry weight basis and determined using the AOAC Official Methods of Analysis (AOAC, 1999) [8] (moisture by oven-drying, crude protein by the Kjeldahl method using a nitrogen conversion factor of 6.25, crude fat by Soxhlet extraction, ash by muffle furnace incineration, and carbohydrate calculated by difference). Proximate analysis indicated that the rice bran consisted of carbohydrates (54.56%), fats (16.57%), and proteins (12.36%). A commercial subtilisin-type protease derived from *Bacillus licheniformis* (Lot I30602b, EC.3.4.21.14; >8 tyrosine-equivalent units mg^−1^) was purchased from the Megazyme (Wicklow, Ireland). α-Chymotrypsin from bovine pancreas (Type II; Lot#SLBT5554, Lyophilized powder, ≥40 Unit mg^−1^ protein) was obtained from Sigma-Aldrich (St. Louis, MO, USA). All other reagents were of analytical grade.

### 2.2. Preparation of Rice Bran Protein, Protein Hydrolysates, and Peptide Fractions

Rice bran protein (RBP) was isolated using an alkaline solubilization-acid precipitation approach with minor adjustments to previously reported protocols by Noptana and Onsaard [9]. RB was subjected to steam treatment (90 °C for 15 min) to inactivate endogenous lipase, followed by removal using hexane. Defatting was performed using commercial-grade hexane. This process was repeated three times to improve the efficiency of oil removal, with each extraction conducted for 1 h at room temperature (25 °C) by stirring at 500 rpm using a solvent-to-solid ratio of 5:1 *v*/*w*. After each cycle, the solvent was removed by decantation, and fresh hexane was added. Residual hexane was eliminated by fume hood evaporation for 2 h and evaporated by oven-drying for 2 h at 55 °C.

The defatted rice bran (DRB) was dispersed in distilled water (1:20, *w*/*v*), adjusted to pH 9 with 2 M NaOH, stirred for 1 h, maintained at pH 9, and centrifuged at 28,313× *g* at 4 °C for 15 min to collect the supernatant. The pH of the supernatant was adjusted to 4.5 using 2 M HCl for 30 min and centrifuged at 28,313× *g* for 15 min at 4 °C. The recovered protein was neutralized, freeze-dried and stored prior to use. Proximate composition of RBP was determined using AOAC methods (1999) and reported on a dry weight basis. Carbohydrate content was calculated by difference.

Protein hydrolysates were generated by enzymatic digestion using *B. licheniformis* and α-chymotrypsin, which were designated as RBPH-B and RBPH-C, respectively, following the method of Onsaard et al. [6].

RBP dispersions were prepared at a ratio of 1:20, *w*/*v* in 50 mM of phosphate buffer (pH 8 for *B. licheniformis* and pH 7.5 for α-chymotrypsin). Enzyme hydrolysis was performed at a fixed enzyme-to-substrate (E/S) ratio of 2.88 U/g protein under continuous stirring. The pH of the protein dispersion was initially adjusted to the optimal pH for each enzyme using the respective buffer systems prior to enzyme addition and was maintained by buffer capacity during hydrolysis without active pH-stat control. Hydrolysis was conducted under controlled temperature conditions (60 °C for *B. licheniformis* and 50 °C for α-chymotrypsin) for a predetermined time interval (30 min to 12 h). At the end of the hydrolysis period, enzyme activity was terminated by thermal treatment at 95 °C for 15 min.

The samples were then cooled in cold tap water at room temperature, followed by centrifugation at 28,313× *g* for 15 min at 4 °C to remove insoluble material. Protein fractionations were performed using tangential-flow ultrafiltration (KrosFlo^®^ KR2i, Spectrum Labs, MA, USA). Ultrafiltration of the protein hydrolysate was performed using a modified polyethersulfone (mPES) membrane with sequential molecular weight cut-off (MWCO) of 100, 10, and 1 kDa and an effective filtration area of 155, 155, and 370 cm^2^. The process was conducted in a cross-flow system at a transmembrane pressure of 2 bar, a crossflow rate of 20 mL min^−1^, and a controlled temperature of 5–10 °C. Sequential ultrafiltration was performed using membranes with molecular weight cut-offs (MWCOs) of 100, 10, and 1 kDa. The 100 kDa retentate was designated as F1, the 10 kDa retentate obtained from the 100 kDa permeate as F2, the 1 kDa retentate obtained from the 10 kDa permeate as F3, and the final 1 kDa permeate as F4. All fractions were freeze-dried and stored at −20 °C for further analysis.

### 2.3. Physicochemical Characterization

#### 2.3.1. Color Attributes

Color parameters (L*, a*, b*) of protein samples and peptide fractions were measured using a colorimeter (UltraScan^®^ Vis, HunterLab, VA, USA). The instrument was calibrated using a light trap and a white-surface calibration plate. Measurement conditions were set to reflectance specular excluded (RSEX) mode, with an area view of 0.375 inches, and nominal UV fitter position. The CIELab color values were recorded as L* (lightness to darkness), a* (redness to greenness), and b* (yellowness to blueness). The total color difference (ΔE) relative to native RBP (control) was calculated using the following equation:

ΔE = (ΔL*^2^ + Δa*^2^ + Δb*^2^)^1/2^

#### 2.3.2. Degree of Hydrolysis

Protein hydrolysis was quantified based on proportion of peptides soluble in 10% trichloroacetic acid (TCA), following the method of Qi et al. [10]. An aliquot of the hydrolysate was mixed with 10% (*w*/*v*) TCA at a sample-to-TCA ratio of 1:1 (*v*/*v*) to obtain a final TCA concentration of 5% (*w*/*v*). The mixture was incubated at room temperature for 30 min to allow precipitation of insoluble proteins, followed by centrifugation at 10,000× *g* for 15 min at 4 °C. The protein content of the TCA-soluble supernatant was determined using the Lowry method [11], and results were expressed as the percentage of TCA-soluble nitrogen relative to total protein.

#### 2.3.3. Protein Content

Total protein concentration of hydrolysates was measured using the Lowry method [11], with bovine serum albumin (BSA) as the calibration standard. Samples were fully solubilized under alkaline solution (4 M NaOH) for 1 h prior to analysis. It is acknowledged that the Lowry assay response is influenced by aromatic amino acid content and peptide chain length; therefore, the values obtained were used for relative comparison among samples rather than for absolute protein quantification.

#### 2.3.4. Surface Hydrophobicity

Surface hydrophobicity (S_0_) was evaluated using the method of Singh et al. [1] with 8-anilino-1-naphthalenesulfonic acid (ANS) as a fluorescent probe. Protein samples and their fractions were prepared at concentrations ranging from 0.02 to 0.10 mg/mL in 10 mM phosphate buffer (pH 7.0). An aliquot of ANS solution (20 μL of 8 mM ANS prepared in 0.1 M phosphate buffer) was added to 4 mL of each diluted sample. The mixtures were gently mixed and incubated at room temperature (25 °C) for 15 min in the dark prior to measurement. Fluorescence intensity was recorded using a spectrofluorometer at an excitation wavelength of 390 nm and an emission wavelength of 470 nm. Surface hydrophobicity (S_0_) was calculated from the initial slope of the linear regression of fluorescence intensity versus protein concentration (mg/mL).

#### 2.3.5. Molecular Weight Distribution

The molecular weights of the RBP, RBPH-B, RBPH-C, and their peptide fractions were analyzed by sodium dodecyl sulfate polyacrylamide gel electrophoresis (SDS-PAGE), following the method of Laemmli [12]. A 12% separating gel and 4% stacking gel were prepared using a Mini-Protein 3 cell (Bio-Rad Laboratories Inc, Richmond, CA, USA). Protein samples (8 mg/mL) were mixed at a 1:1 (*v*/*v*) ratio with sample buffers (0.5 M Tris-HCl, pH 6.8, 20% glycerol, 10% SDS, and 0.2% (*w*/*v*) bromophenol blue), with and without 2-mercaptoethanol, and boiled for 3 min. Seven microliters of the protein solution and five microliters of molecular weight marker (BIO-HELIX-PM008-0500, GenScript, Keelung, Taiwan) were loaded per well. Electrophoresis was conducted at a constant voltage of 200 V. The gel was stained overnight in 0.3% (*w*/*v*) Coomassie brilliant blue R-250 mixed in 45% (*v*/*v*) methanol and 10% (*v*/*v*) acetic acid. Destaining was performed sequentially using 50% (*v*/*v*) ethanol and 7.5% (*v*/*v*) acetic acid solutions for 3 h. The gel was then dried and scanned for band intensity and distribution, which were used to estimate the molecular weights of the different samples.

#### 2.3.6. Secondary Structure Analysis

Fourier transform infrared (FT-IR) spectroscopy (Nicolet 6700, Thermo Scientific, MA, USA) was used to analyze the functional groups and secondary structure of the proteins and peptides, according to the method of Onsaard et al. [6], with slight modifications. IR spectra were recorded between 4000 and 400 cm^−1^ with 32 scans per sample at a resolution of 6 cm^−1^ under ambient conditions. Secondary-structure analysis was performed on the amide I region (1600–1700 cm^−1^). Second-derivative spectra were calculated to identify peak positions, followed by Gaussian curve fitting using PeakFit software (Version 4.12, Systat Software Inc., San Jose, CA, USA). The number of component bands was fixed based on second-derivative minima. Wavenumber assignments were as follows: α-helix (1650–1658 cm^−1^), β-sheet (1610–1640 and 1680–1695 cm^−1^), β-turn (1660–1680 cm^−1^), and random coil (1640–1650 cm^−1^). The relative proportion of each secondary-structure component was calculated from the integrated area of the corresponding fitted bands.

### 2.4. Total Phenolic Content

Total phenolic content of the samples was determined using the Folin–Ciocalteu method, according to a previously described method [13,14,15], with slight modifications. Briefly, 0.2 g of sample was stirred with 4 mL of 80% ethanol for 30 min, centrifuged at 3000 rpm for 10 min at 4 °C, and then the supernatant was collected and stored at 8 °C. An aliquot (100 μL) was mixed with 2 mL of 2% sodium carbonate solution. After 2 min, 100 μL of 50% (*v*/*v*) Folin–Ciocalteu’s reagent was added, and the mixture was incubated in the dark at room temperature for 30 min. The absorbance was then measured at 750 nm using a UV–visible spectrophotometer. A standard curve was prepared using gallic acid (0–0.4 mg/mL). The total phenolic content was then calculated using the following formula:

C = cV/m

Here, C is the total phenolic content (mg GAE/g dw), c is the concentration of gallic acid from the calibration curve (mg/mL), *V* is the volume of the extract (mL), and *m* is the mass of the sample (g).

### 2.5. Antioxidant Activity Assays

The antioxidant properties of the proteins and peptides were evaluated using multiple assays.

#### 2.5.1. ABTS Radical Scavenging Activity

The ABTS assay was used to determine the radical scavenging activity of the different samples, which was conducted according to the method described previously [16,17]. The ABTS^+^ stock solution was prepared by mixing 7 mM ABTS with 2.45 mM potassium persulfate at a volume ratio of 1:0.5 (*v*/*v*) and incubating the mixture in the dark for 12–16 h. The solution was then diluted with phosphate-buffered saline (PBS, pH 7.4) solution to an absorbance of 0.700 ± 0.02 at 734 nm (cm^−1^ path length). After dilution, 25 µL of peptide fraction (0.5–6 mg/mL) or distilled water (control) was mixed with 2.5 mL of ABTS^+^ solutions and incubated at room temperature for 6 min. Ascorbic acid (0.01–0.4 mg/mL) was used as a positive control. The absorbance of the solutions was measured at 734 nm using a UV–visible spectrophotometer, and the radical scavenging activity was then calculated as

ABTS radical scavenging (%) = [(A_control_ − A_sample_)/A_control_] × 100

Here, A_control_ and A_sample_ are the absorbances of the control and sample, respectively.

EC_50_ values for ABTS•^+^ radical-scavenging assays were determined by constructing dose–response curves using at least five sample concentrations spanning the linear and nonlinear regions of inhibition.

#### 2.5.2. DPPH Radical Scavenging Activity

The DPPH assay was performed according to a method described previously [18,19]. Aqueous sample solutions (0.1–1 mg/mL) or positive control (ascorbic acid, 0.5–50 μg/mL) were mixed with an equal volume of 100 μM DPPH solution prepared in methanol (1:1, *v*/*v*), resulting in a final methanol content of 50% (*v*/*v*). Under these conditions, no visible precipitation or turbidity was observed. The reaction mixtures were incubated in the dark at room temperature for 30 min, and absorbance was measured at 517 nm using a UV–visible spectrophotometer. Sample blanks containing the same solvent composition without DPPH were used for background correction to account for any baseline absorbance or light scattering. The DPPH radical-scavenging activity was calculated as follows:

DPPH radical scavenging (%) = [(A_control_ − (A_sample_ − A_background_))/A_control_] × 100

Here, A_control_, A_sample_, and A_background_ represented the absorbance of the control, sample, and background, respectively. EC_50_ values were determined from dose–response curves constructed using at least five concentrations spanning the linear and nonlinear regions of inhibition.

#### 2.5.3. Metal Chelating Activity

The metal chelating activity of the samples was determined following a method described previously [20]. One milliliter of peptide solution (1 mg/mL) or positive control (1 mg/mL ascorbic acid) was mixed with 3.7 mL of distilled water, following by the addition of 0.1 mL of 2 mM ferrous chloride (FeCl_2_) solution and 0.2 mL of 5 mM ferrozine solution. After vertexing and incubation for 10 min at room temperature, the absorbance was measured at 562 nm using a UV–visible spectrophotometer. The results were then expressed as mmol EDTA equivalent/g sample.

#### 2.5.4. Linoleic Peroxidation Inhibition Assay

The linoleic acid peroxidation inhibition assay was performed according to a method described previously [21]. Protein fractions and the positive control (ascorbic acid) were prepared at a final concentration of 2 mg/mL. For each assay, 1.0 mL of sample solution was mixed with 130 μL of linoleic acid and 10 mL of ethanol, and the total volume was adjusted to 25 mL with distilled water. The negative control consisted of the same reaction mixture without the addition of protein fractions or antioxidant compounds. Reaction mixtures were incubated at 42 °C in the dark for 24 h under ambient air conditions, without oxygen purging or headspace control, and without agitation to allow spontaneous oxidation of linoleic acid. After incubation, 0.1 mL of the reaction mixture was added to 4.7 mL of 75% (*v*/*v*) ethanol, followed by the addition of 0.1 mL of 30% (*w*/*v*) ammonium thiocyanate, and 0.1 mL of 20 mM ferrous chloride. The mixture was incubated for 3 min at room temperature, and the absorbance was measured at 500 nm using a UV–visible spectrophotometer. The inhibition was then calculated as

Inhibition (%) = [(A_control_ − A_sample_)/A_control_] × 100

Here, A_control_ and A_sample_ are the measured absorbances of the control and sample, respectively.

### 2.6. Statistical Analysis

All experiments were conducted in three independent experimental runs, with each measurement performed in triplicate. Data are therefore reported as mean ± standard deviation (n = 9). Statistical significance was analyzed using one-way analysis of variance (ANOVA), followed by Duncan’s multiple range test at *p* < 0.05, using SPSS software (Version 15.0, SPSS Inc., Chicago, IL, USA).

## 3. Results and Discussion

### 3.1. Characteristics of Rice Bran Protein, Rice Bran Hydrolysates, and Peptide Fractions

**Figure 1:**
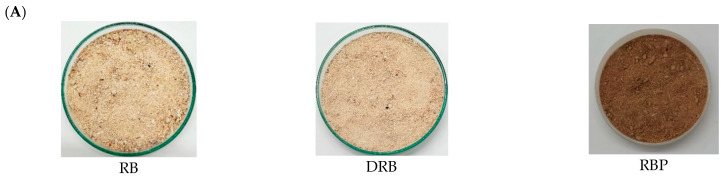

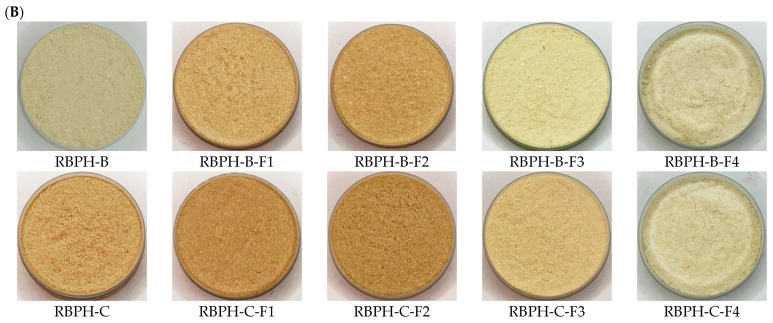
Visual appearance of rice bran and its processed derivatives: (**A**) Rice bran (RB) and its derivatives: defatted rice bran (DRB), and rice bran protein (RBP), (**B**) rice bran protein hydrolysates produced by *Bacillus licheniformis* (RBPH-B) and α-chymotrypsin (RBPH-C), along with their peptide fractions obtained by ultrafiltration. Protein fractions were classified as follows: F1 (>100 kDa), F2 (10–100 kDa), F3 (1–10 kDa), and F4 (<1 kDa).

After rice protein extraction, the chemical compositions of the rice bran proteins (RBPs) were determined: 72.06% protein, 0.80% fat, 5.14% ash, 1.16% moisture, and 20.84% carbohydrate. The visual appearances of the rice bran (RB) and its processed derivatives, defatted rice bran (DRB), and rice bran protein (RBP), are shown in Figure 1A, while the appearances of the rice bran protein hydrolysates produced by *Bacillus licheniformis* (RBPH-B) and α-chymotrypsin (RBPH-C), along with their peptide fractions obtained by ultrafiltration, are presented in Figure 1B. Defatting resulted in a lighter color compared to the native RB, whereas the extracted RBP exhibited a darker appearance, likely due to the increased concentration and to interactions with residual phenolic compounds [22]. These interactions can lead to the formation of protein–phenolic complexes, which may influence the visual characteristics of protein extracts. However, alternative factors, such as alkaline-induced oxidation reactions and potential Maillard-type reactions between residual carbohydrates and amino groups during protein isolation, may also contribute to color development. In the absence of direct phenolic profiling or browning index measurements, these effects should be considered as plausible contributors rather than definitive causes. Following enzymatic hydrolysis and ultrafiltration, the peptide fractions displayed progressively lighter colors, with the <1 kDa fractions (F4) appearing the lightest. These visual changes suggest extensive protein breakdown and the removal of pigmented complexes during hydrolysis and fractionation [23].

#### 3.1.1. Color Characteristics

The color characteristics of RBP, RBPH-B, RBPH-C, and their peptide fractions are shown in Table 1, while the overall appearance is shown in Figure 1. The lightness (L*), redness (a*), and yellowness (b*) values of the RBP were 54.14, +4.40, and +13.03, respectively, indicating that they had a reddish-yellowish appearance. After enzymatic hydrolysis and ultrafiltration, the color values of RBPH-B, RBPH-C, and their protein fractions changed, as indicated by an increase in lightness and yellowness and a decrease in redness. The smaller peptides (RBPH-B-F4 and RBPH-C-F4) exhibited the highest L* and b* values, and lowest a* value. These results suggested that the lightness of the powdered RBP increased after hydrolysis and ultrafiltration, which may have been due to the formation of smaller particles in the powder during freeze drying, as well as removal of pigments, leading to stronger light scattering and weaker light absorption [5]. The observed increase in yellowness and decrease in redness may be attributed to the degradation of flavonoids and other polyphenols [24], as well as the removal of pigmented complexes during hydrolysis and fractionation [23]. Furthermore, RBPH-C and its protein fractions exhibited higher lightness compared to RBPH-B and its protein fractions, while the RBPH-B samples showed greater redness and yellowness. Among all samples, RBPH-C-F4 exhibited the highest lightness, while RBPH-B and certain fractions (F1–F2) had the highest redness, and RBPH-B-F4 showed the highest yellowness.

In addition, the ΔE* value increased following hydrolysis and ultrafiltration, indicating an overall increase in color change. This difference may be explained by the hydrolysis conditions; RBPH-B samples were processed under higher temperature, higher pH, and longer hydrolysis times compared to RBPH-C, leading to more intense redness and yellowness. These finding are consistent with the report of Arsa and Puechkamutr [25], who observed that a higher pH promoted melanoidin formation and deprotonation of amino acids, thereby enhancing their reactivity with reducing sugars (Maillard reaction).

#### 3.1.2. Degree of Hydrolysis and Protein Content

**Figure 2:**
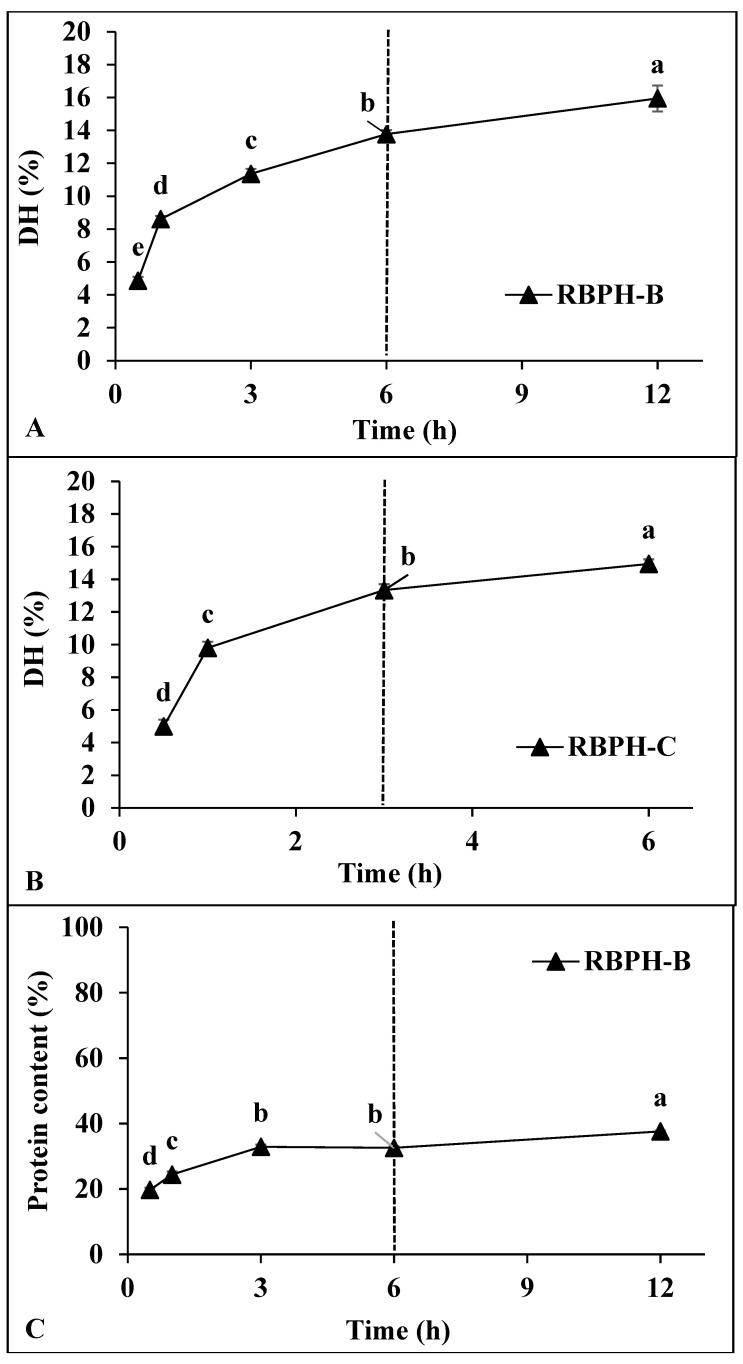

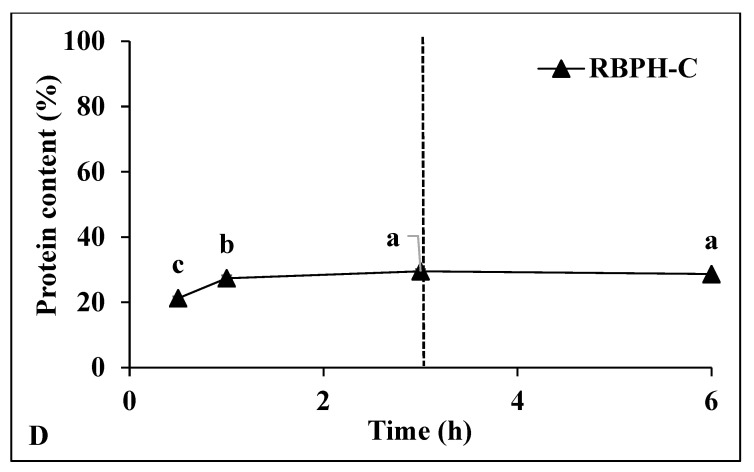
Degree of hydrolysis (DH) and protein content (PC) of rice bran protein hydrolysate over time at an enzyme concentration of 2.88 U/g protein. (**A**) DH of rice bran protein hydrolysate produced using *Bacillus licheniformis* (RBPH-B) over a hydrolysis period of 0–12 h. (**B**) DH of rice bran protein hydrolysate produced using α-chymotrypsin (RBPH-C) over a hydrolysis period of 0–6 h. (**C**) PC of rice bran protein hydrolysate produced using *Bacillus licheniformis* (RBPH-B) over a hydrolysis period of 0–12 h. (**D**) PC of rice bran protein hydrolysate produced using α-chymotrypsin (RBPH-C) over a hydrolysis period of 0–6 h. Different lowercase letters indicate significant differences (*p* < 0.05).

The degree of hydrolysis (DH) of RBP was evaluated after enzymatic treatment with protease from *Bacillus licheniformis* (RBPH-B) and α-chymotrypsin (RBPH-C) at an enzyme concentration of 2.88 U/g protein over different hydrolysis periods (0–12 h for RBPH-B and 0–6 h for RBPH-C) (Figure 2A,B). Although α-chymotrypsin required a shorter hydrolysis time than *Bacillus licheniformis,* the resulting DH values were comparable. The DH of RBPH-B and RBPH-C increased from 4.86% to 15.95% and from 4.99% to 14.95%, respectively, with extended hydrolysis times. A rapid increase in DH was observed during the early stages (1–3 h for RBPH-B and 0–1 h for RBPH-C), followed by a slower increase thereafter. These results suggest that α-chymotrypsin cleaved peptide bonds more rapidly than the *Bacillus licheniformis* proteases, likely because α-chymotrypsin can hydrolyze peptide bonds at both the carboxyl side of aromatic amino acids and internal bonds, while *Bacillus licheniformis* protease primarily only targets internal peptide bonds [6]. Nevertheless, after 6 h for RBPH-B and 3 h for RBPH-C, the DH value converged to approximately 13.77% and 13.34%, respectively (Figure 2A,B). This result is consistent with that of previous studies, which found that protein hydrolysates with a DH of around 13% often display enhanced antioxidant properties [5]. Therefore, hydrolysis periods of 6 h for RBPH-B and 3 h for RBPH-C were selected to produce protein hydrolysates and their respective protein fractions for further analysis, respectively.

The impact of enzymatic hydrolysis on protein content (PC) is shown in Figure 2C,D. For RBPH-B, the PC increased steadily with increasing hydrolysis time, reaching 19.70%, 24.40%, 32.88%, 32.55%, and 37.63% after 0.5, 1, 3, 6, and 12 h, respectively (Figure 2C). Similarly, the PC of RBPH-C increased to 21.25%, 27.37%, and 29.52% after 0.5, 1, and 3 h, respectively, but plateaued thereafter (Figure 2D). These trends parallel the DH profile and suggest that enzymatic hydrolysis enhances protein content by breaking down large protein molecules into smaller, more soluble peptides. This finding is consistent with earlier reports that protein hydrolysates produced by enzymatic treatment generally show improved water solubility due to their reduced molecular size and increased exposure of hydrophilic groups [1,5,26]. The improvement in solubility is crucial for the potential application of rice bran-derived peptides as functional ingredients in aqueous-based food and beverage systems.

#### 3.1.3. Surface Hydrophobicity

**Figure 3:**
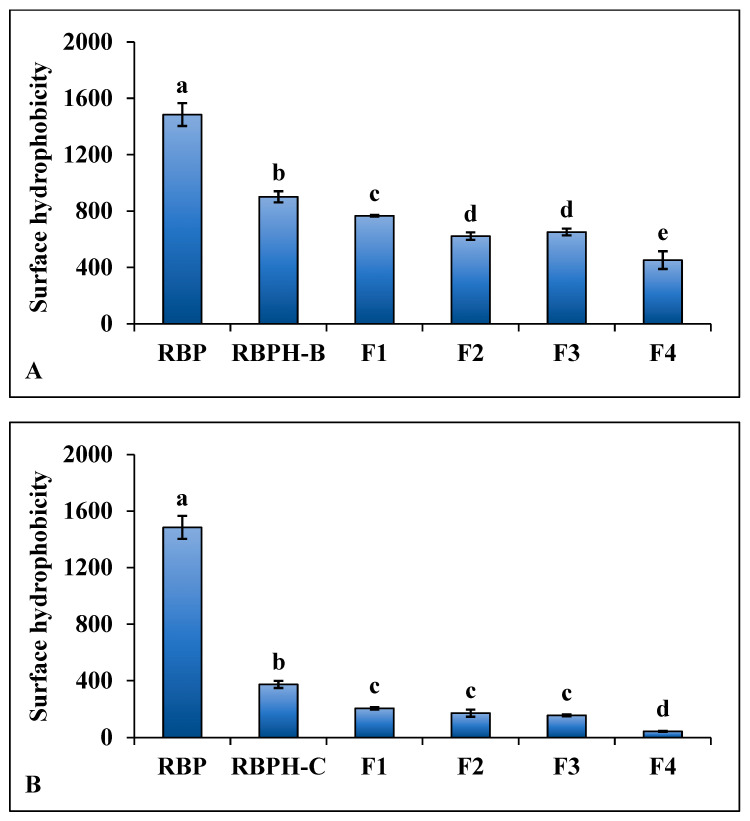
Surface hydrophobicity (S_0_) of rice bran protein (RBP), rice bran protein hydrolysates, and their protein fractions at protein concentrations ranging from 0.02 to 0.1 mg/mL: (**A**) RBP, rice bran protein hydrolysate produced using *Bacillus licheniformis* (RBPH-B), and their protein fractions (F1–F4). (**B**) RBP, rice bran protein hydrolysate produced using α-chymotrypsin (RBPH-C), and their protein fractions (F1–F4). Different lowercase letters indicate significant differences (*p* < 0.05) in S_0_ among proteins, hydrolysates, and protein fractions.

The surface hydrophobicity (S_0_) of proteins reflects the extent of exposure of non-polar regions on their surfaces to the surrounding aqueous phase [27]. The measured S_0_ values for RBPH-B and RBPH-C were compared to that of RBP (Figure 3A,B). Both hydrolysates exhibited significantly lower S_0_ values than RBP, suggesting substantial structural modifications during hydrolysis. Notably, RBPH-C had a lower S_0_ value than RBPH-B, suggesting that it was less hydrophobic. Additionally, ultrafiltration resulted in progressively decreasing S_0_ values across peptide fractions, with the <1 kDa fractions (RBPH-B-F4 and RBPH-C-F4) showing the lowest S_0_ levels. These trends indicate that hydrolysis of rice bran proteins led to the formation of smaller, more hydrophilic peptides. Changes in surface hydrophobicity appeared to coincide with variations in secondary-structure features, particularly α-helix content, across different hydrolysates and molecular weight fractions. However, this observation is descriptive in nature, and no statistical correlation analysis was performed. Therefore, any relationship between surface hydrophobicity and secondary-structure composition should be interpreted qualitatively rather than as a validated correlation.

The lower S_0_ value observed for RBPH-C compared to RBPH-B may be attributed to enzyme specificity, as α-chymotrypsin preferentially cleaves bonds near aromatic residues, generating smaller and more hydrophilic peptides than *Bacillus licheniformis* protease. The progressive decrease in S_0_ across protein fractions reflects the removal of large hydrophobic structures during hydrolysis and size fractionation. Smaller peptides with fewer non-polar regions contribute to higher water solubility and less aggregation in aqueous systems, essential features for many food applications. Our observations align with previous studies on peptides derived from other plant proteins, including those from kidney bean [28], sesame [5], and soybean [29]. However, contrasting results were reported by Kaprasob et al. [30] for king boletus mushroom peptides, where <1 kDa fractions had higher S_0_ values, highlighting the influence of protein source, enzyme specificity, and hydrolysis and fractionation conditions on the hydrophobic character of the peptides produced [1].

Overall, the observed decrease in surface hydrophobicity after enzymatic hydrolysis and ultrafiltration indicates that rice bran proteins can be converted into more hydrophilic peptides, which may lead to enhanced water solubility, thereby expanding their applications in functional foods and beverages.

#### 3.1.4. SDS-PAGE Analysis

**Figure 4:**
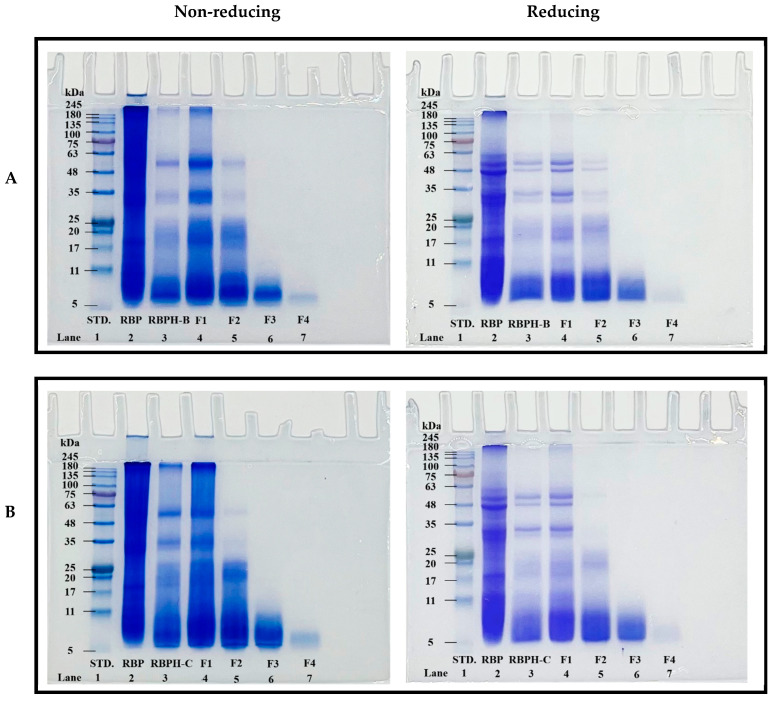
SDS-PAGE patterns of rice bran protein (RBP), rice bran protein hydrolysates produced by *Bacillus licheniformis* (RBPH-B) and α-chymotrypsin (RBPH-C), and their corresponding protein fractions (F1–F4) under non-reducing and reducing conditions. (**A**) SDS-PAGE patterns of RBP, RBPH-B, and their peptide fraction (F1–F4). (**B**) SDS-PAGE patterns of RBP, RBPH-C, and their peptide fraction (F1–F4). Lane assignments: 1. Molecular weight standard (STD), 2. RBP, 3. RBPH, 4. F1 (>100 kDa), 5. F2 (10–100 kDa), 6. F3 (1–10 kDa), and 7. F4 (<1 kDa).

The SDS-PAGE profiles of RBP, RBPH-B, RBPH-C, and their protein fractions (F1–F4) were evaluated under non-reducing and reducing conditions (Figure 4). Native RBP exhibited a wide range of bands between 7 and 167 kDa, with three major protein bands observed at <17, 31–39, and >55 kDa under both conditions (lane 2, Figure 4A,B). These bands likely correspond to prolamin, glutelin (acidic subunits), and pro-glutelin, respectively. After enzymatic hydrolysis, the band intensities of RBPH-B, RBPH-C, and their protein fractions changed in a manner that was consistent with the formation of lower molecular weight fractions. Under non-reducing conditions, prominent bands were still visible above 65 kDa for RBPH-B, RBPH-C, and their F1 fractions, whereas under reducing conditions, these bands largely disappeared. This indicates that the reducing agent (2-mercaptoethanol) disrupted disulfide bonds, releasing lower molecular weight fractions. The ultrafiltration process further altered the protein profiles, showing that smaller peptides passed through the membranes, particularly in fractions F3 and F4, resulting in faint or absent bands at higher molecular weights. The faint or absent bands observed in the F3 and F4 fractions should be interpreted with caution. Conventional 12% SDS-PAGE has limited resolution and detection sensitivity for peptides below approximately 3 kDa, and therefore the lack of visible bands does not confirm that these fractions consist exclusively of <1 kDa peptides. Instead, these results reflect methodological limitations of SDS-PAGE for low-molecular-weight species, and more suitable analytical techniques would be required for definitive size characterization. The SDS-PAGE results confirmed that the hydrolysates contained a mixture of different peptides, mainly within the molecular weight ranges previously reported by Jayaprakash et al. [31] and Xia et al. [32], who identified albumin (15–56 kDa), prolamin (12–17 kDa), globulin (18–26 kDa), glutelin (19–23 and 30–40 kDa for basic and acidic subunits, respectively), and pro-glutelin (>55 kDa). Similar molecular weight distributions were also noted by Onsaard et al. [6] and Fathi et al. [2], although Phongthai et al. [3] observed a slightly narrower range.

Enzymatic hydrolysis by both proteases led to a visible degradation of the RBPs, as indicated by the reduced band intensities and lower molecular weights of the protein fractions present. The differences in protein patterns between non-reducing and reducing conditions further highlighted the importance of disulfide bonds in maintaining the structural integrity of RBP and its peptides. Disulfide bond cleavage facilitated by reducing agents exposed smaller polypeptides that were otherwise masked in non-reducing conditions. Notably, for both enzymes, the SDS-PAGE patterns of RBPH-B and RBPH-C were similar. This observation can be attributed to the comparable degree of hydrolysis (~13%) achieved in both treatments, suggesting that DH strongly influenced the molecular size distribution regardless of protease specificity. The ultrafiltration results align with findings from previous studies on sesame protein hydrolysates [5] and soy protein hydrolysates [29], where membrane fractionation effectively separated peptides based on size, leading to the disappearance of higher molecular weight bands in the lower molecular weight fractions.

Overall, enzymatic hydrolysis combined with membrane ultrafiltration effectively reduced the molecular weight of rice bran-derived peptides, contributing to enhanced functional properties such as solubility and antioxidant activity. However, conventional 12% Laemmli SDS-PAGE has limited resolving power for peptides below approximately 3 kDa; therefore, weak or absent bands in low-molecular-weight fractions should be interpreted with caution. More suitable techniques, including tricine SDS-PAGE, size-exclusion chromatography (SEC), or LC–MS/MS, are required for accurate resolution and quantification of peptides in the <1 kDa range.

#### 3.1.5. Protein Secondary Structures

**Figure 5:**
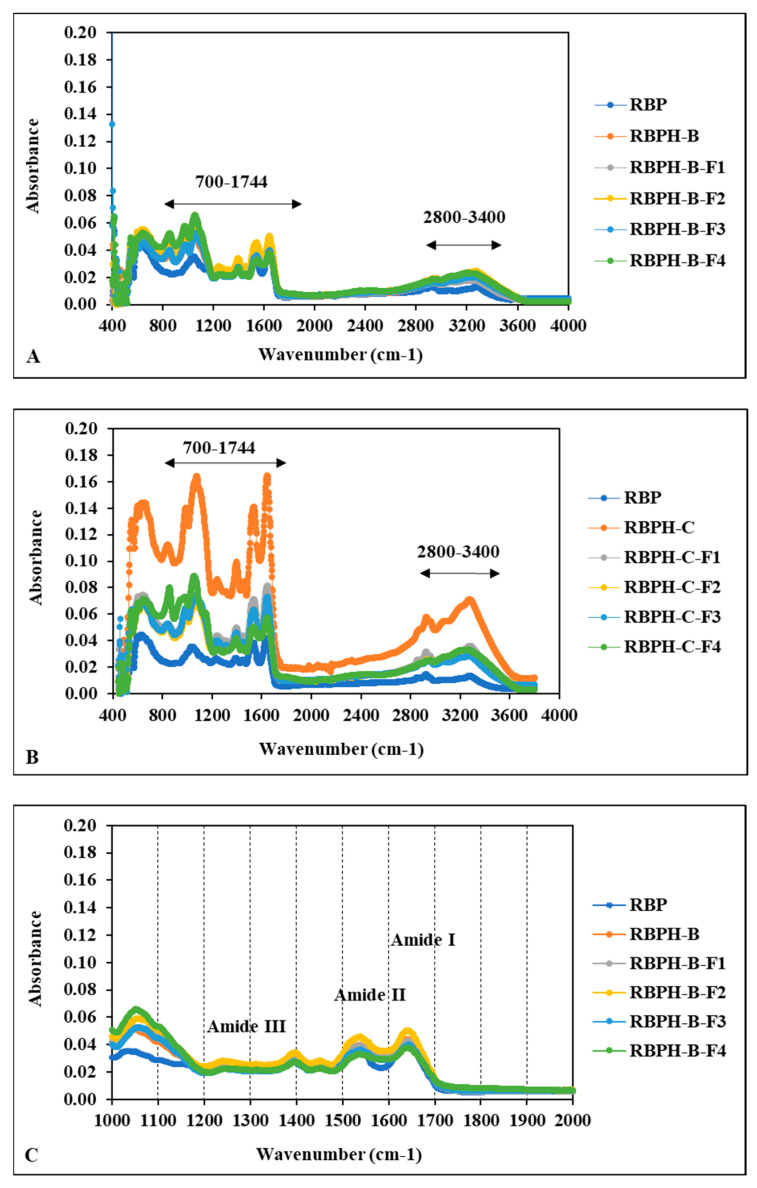

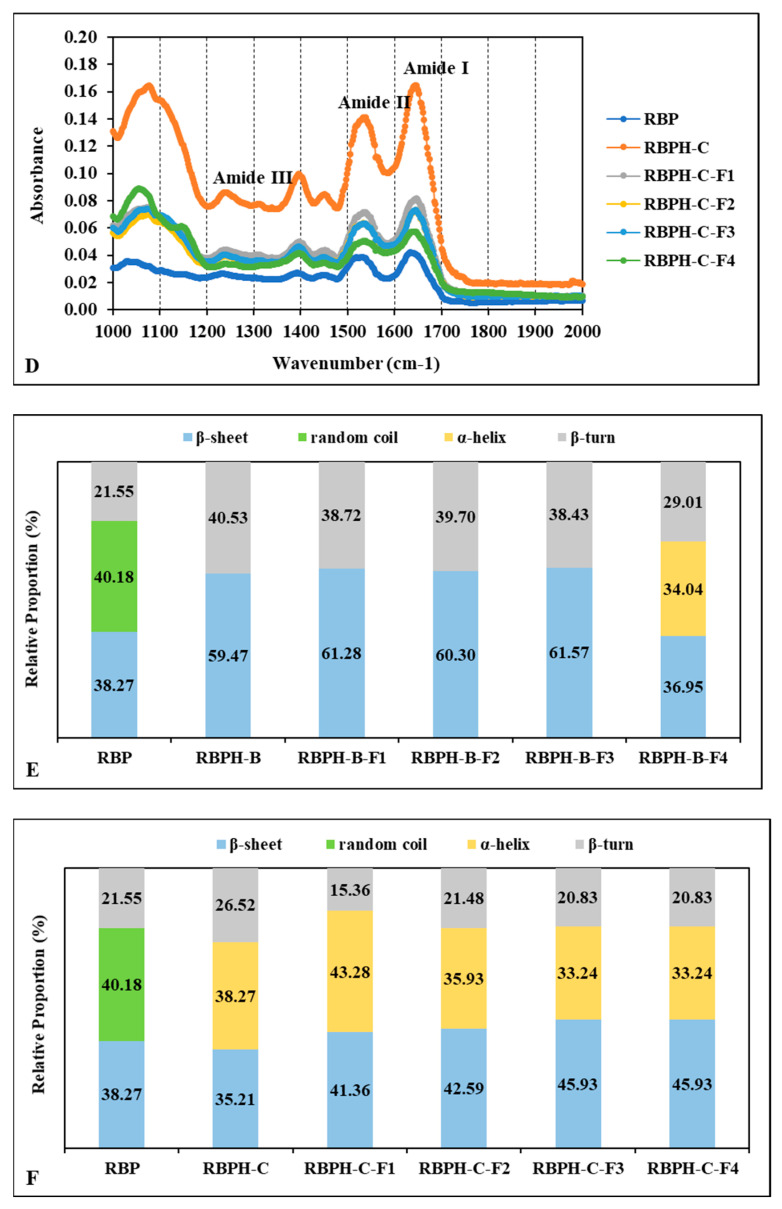
Fourier transform infrared (FT-IR) spectroscopy analysis of rice bran protein (RBP), rice bran protein hydrolysates, and their protein fractions (F1–F4). (**A**) FT-IR spectra of RBP, RBPH-B (hydrolysates produced using Bacillus licheniformis), and their fractions (F1–F4) over the wavenumber range of 4000–400 cm^−1^. (**B**) FT-IR spectra of RBP, RBPH-C (hydrolysates produced using α-chymotrypsin), and their fractions (F1–F4) over the wavenumber range of 4000–400 cm^−1^. (**C**) FT-IR spectra of RBP, RBPH-B, and their fractions (F1–F4) focusing on amide I, II and III bands in the wavenumber range of 1000–2000 cm^−1^. (**D**) FT-IR pattern of RBP, RBPH-C, and their protein fractions (F1–F4) focusing on amide I, II and III bands in the wavenumber range of 1000–2000 cm^−1^. (**E**) Relative proportion of secondary structure components (β-sheet, α-helix, β-turn, and random coil) in RBP, RBPH-B, and their fractions (F1–F4). (**F**) Relative proportion of secondary structure components (β-sheet, α-helix, β-turn, and random coil) in RBP, RBPH-C, and their fractions (F1–F4).

Fourier transform infrared (FTIR) spectroscopy was used to determine the secondary structures of RBP, RBPH-B, RBPH-C, and their protein fractions (F1–F4) (Figure 5). The FTIR spectra (Figure 5C,D) displayed characteristic amide regions, amide I (1600–1700 cm^−1^), II (1500–1600 cm^−1^), and III (1200–1400 cm^−1^) bands, corresponding to C=O stretching, C-N stretching, and N-H bending vibrations, respectively [33].

In RBP, the sharp amide I absorption peak appeared at 1639 cm^−1^, while amide II and III bands were observed at 1525 and 1391 cm^−1^, respectively. After enzymatic hydrolysis and ultrafiltration, shifts in these peak positions were noted for RBPH-B, RBPH-C, and their protein fractions, indicating conformational changes in the protein backbone. For instance, RBPH-B and its fractions exhibited slight shifts in amide I (1640–1642 cm^−1^) and amide II (1534–1550 cm^−1^) regions (Figure 5C), while RBPH-C and its fractions exhibited shifts in its amide I (1643–1645 cm^−1^) and amide II (1531–1534 cm^−1^) bands (Figure 5D). These modifications were more pronounced in the RBPH-C samples, suggesting greater structural alterations.

Quantitative analysis of secondary structure compositions (Figure 5E,F) revealed that RBP was primarily composed of β-sheet (38.27%), random coil (40.18%), and β-turn (21.55%) regions, with no detectable α-helix regions. Upon hydrolysis, RBPH-B and its fractions (F1–F3) exhibited increased β-sheet (59–62%), and β-turn (38–41%) contents, while RBPH-B-F4 exhibited substantial amounts of α-helix (34%) and β-sheet (37%) structures. In contrast, RBPH-C and its fractions contained appreciable β-sheet (35–46%), α-helix (33–43%), and β-turn (15–27%) contents, without detectable random coil regions.

These results demonstrate that enzymatic hydrolysis and ultrafiltration significantly influenced the secondary structure of the rice bran proteins. Shifts in amide I and II bands indicated the disruption and reorganization of hydrogen bonding patterns, resulting in altered conformations. Compared to native RBP, hydrolyzed samples displayed a reduction in random coil structures and an increase in more ordered structures, such as β-sheets and α-helices, particularly in the smaller protein fractions (<1 kDa). The increased β-sheet and β-turn contents observed in RBPH-B are consistent with a previous finding that enzymatic hydrolysis promoted structural ordering [5,27]. Similarly, the emergence of α-helix structures in RBPH-B-F4 and RBPH-C fractions suggests that smaller peptides were more prone to adopting compact and stable helical conformations after size fractionation, which aligns with prior reports by Singh et al. [1] and Onsaard et al. [6]. Furthermore, hydrolysis with α-chymotrypsin resulted in a higher proportion of α-helix-related spectral features compared with hydrolysis using *Bacillus licheniformis* protease, suggesting that enzyme specificity influences the structural characteristics of the resulting peptides. Under the current FTIR curve-fitting model, α-helix contributions were not resolved in native rice bran protein, while random coil components were not resolved in the RBPH-C fractions. These observations should be interpreted cautiously, as FTIR-based secondary-structure estimation depends on fitting constraints and sample state and does not preclude the presence of these structural elements. Nevertheless, an increased contribution of α-helix-related structures may be associated with greater molecular compactness and flexibility, which could potentially enhance peptide solubility and bioactivity.

### 3.2. Total Phenolic Content

The impact of enzymatic hydrolysis and ultrafiltration on the phenolic content of RBP, RBPH-B, RBPH-C, and their protein fractions were evaluated using the Folin–Ciocalteu assay.

**Figure 6:**
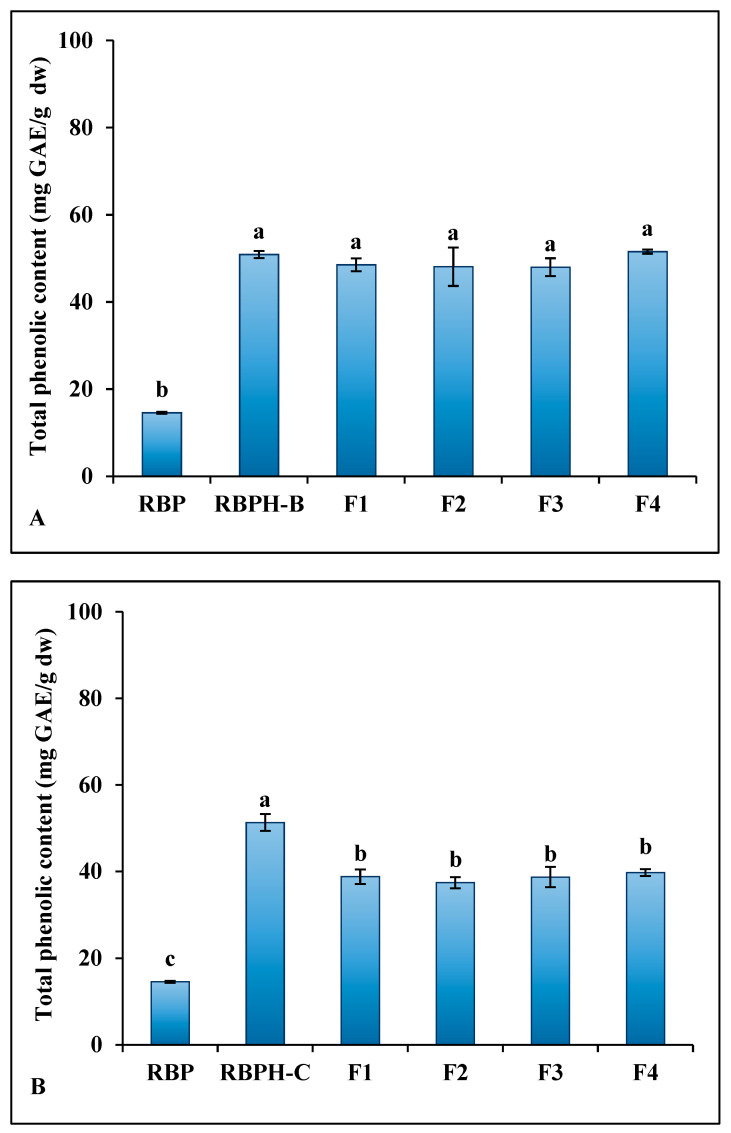
Total phenolic content of rice bran protein (RBP), rice bran protein hydrolysates, and their protein fractions (F1–F4). (**A**) Total phenolic content of RBP, RBPH-B (hydrolysate produced using *Bacillus licheniformis*), and their protein fractions (F1–F4). (**B**) Total phenolic content of RBP, RBPH-C (hydrolysate produced using α-chymotrypsin), and their protein fractions (F1–F4). Different lowercase letters indicate significant differences (*p* < 0.05) among samples.

Quantitative analysis of the total phenolic content (TPC) of the different samples (Figure 6) showed that native RBP had the lowest TPC value (14.57 mg GAE/g dw). Following enzymatic hydrolysis and ultrafiltration, the TPC ranged from around 48 to 52 mg GAE/g dw, with no significant differences among fractions (Figure 6A). The RBPH-C fraction showed the highest TPC (51.32 mg GAE/g dw), while its protein fractions exhibited slightly lower, but comparable, values ranging from 37 to 40 mg GAE/g dw (Figure 6B).

The marked increase in TPC value after enzymatic hydrolysis indicates that proteolysis facilitates the release of phenolic compounds or phenolic-rich peptides from of rice bran proteins. This result is consistent with prior studies demonstrating that hydrolysis liberates phenolic amino acids, such as tyrosine, which contribute to the antioxidant properties of peptides [34,35]. The higher TPC observed in the hydrolysates compared to the RBP suggests that the enzyme treatments enhanced the release of phenolic groups. Between the two enzymes, α-chymotrypsin-treated samples (RBPH-C) generally exhibited higher TPC than *Bacillus licheniformis* protease-treated samples (RBPH-B), suggesting that enzyme specificity affects the release of phenolic residues during hydrolysis.

Notably, a reduction in TPC was observed after ultrafiltration of the hydrolysates. This change may be due to the partial loss of phenolic compounds through membrane permeation, as small phenolics and phenolic peptides can pass through ultrafiltration membranes. Our results align with the findings of Castel et al. [13] for ultrafiltration of amaranth peptides. Given that phenolic compounds act as hydrogen donors, reducing agents, and free radicals scavenging [15], the enhancement of TPC through enzymatic hydrolysis supports the potential of rice bran peptides as functional ingredients with antioxidant properties.

### 3.3. Antioxidant Properties

The antioxidant properties of the different peptides were then determined using a number of different assays to assess their potential as plant-derived potential antioxidant ingredients in foods and beverages.

#### 3.3.1. ABTS Radical Scavenging Activity

The ABTS radical scavenging activity of RBPH-B, RBPH-C, and their corresponding protein fractions, was evaluated and compared to ascorbic acid (a positive control). As shown in Table 2, ascorbic acid exhibited the lowest EC_50_ value, confirming its strong antioxidant capacity. In contrast, both RBPH-B and RBPH-C exhibited significantly higher EC_50_ values, reflecting their lower scavenging efficiency. A clear trend was observed in which the EC_50_ values decreased with decreasing molecular weight, indicating enhanced antioxidant activity in the lower-molecular-weight peptide fractions. The EC_50_ values ranged from 3.01 to 1.84 mg/mL for RBPH-B and its fractions, and from 1.99 to 0.94 mg/mL for RBPH-C and its fractions. The <1 kDa fraction derived from α-chymotrypsin hydrolysis (RBPH-C-F4) showed the highest ABTS^+^ scavenging activity among all samples. This inverse correlation between peptide size and ABTS^+^ radical scavenging activity is consistent with previous findings that smaller peptides tend to have greater antioxidant capacities [3,5,36]. These enhancements are likely due to the increased number of exposed phenolic amino acid residues (such as tyrosine and tryptophan) in smaller peptides, allowing for better interaction with ABTS^+^ radicals.

Notably, the protein fractions from RBPH-C could scavenge the ABTS^+^ radicals more effectively than those from RBPH-B. This effect may be attributed to the enzymatic specificity of α-chymotrypsin, which targets aromatic amino acids, thereby producing peptides rich in hydrogen donating groups (H^+^). The greater antioxidant activity may also be related to the lower surface hydrophobicity of the RBPH-C peptide, which increased its dispersibility in aqueous media.

#### 3.3.2. DPPH Radical Scavenging Activity

The antioxidant potential of RBPH-B, RBPH-C, and their protein fractions (F1–F4) were also evaluated using the DPPH radical scavenging assay, and the EC_50_ values are summarized in Table 2. As expected, ascorbic acid (a positive control) displayed the highest antioxidant activity, as indicated by the lowest EC_50_ value. Among the rice bran protein hydrolysates, the smaller protein fractions showed significantly stronger radical scavenging abilities (*p* < 0.05). In particular, RBPH-B-F3, RBPH-B-F4, and RBPH-B-F2 exhibited EC_50_ values around 200 μg/mL, indicating strong DPPH scavenging activity, followed by RBPH-B-F1 (475 μg/mL) and RBPH-B (908 μg/mL). A similar trend was observed for peptides derived from α-chymotrypsin hydrolysis, where RBPH-C-F4 exhibited the highest activity (210 μg/mL), followed by RBPH-C-F3, RBPH-C-F2, and RBPH-C-F1, with RBPH-C showing the lowest activity (399 μg/mL). No significant differences (*p* > 0.05) were observed among the most active smaller protein fractions. These findings support the hypothesis that smaller peptides possess enhanced antioxidant activity due to their increased accessibility to free radicals and the presence of more exposed electron-donating amino acid residues. Our results are consistent with previous studies reporting that peptides with lower molecular weights exhibited stronger DPPH radical scavenging capacity [3,5,37].

#### 3.3.3. Metal Chelating Activity

Transition metals such as iron and copper act as pro-oxidants by catalyzing oxidative reactions. Metal chelation limits their participation in these reactions, thereby contributing indirectly to antioxidant protection. The ferrous ion-chelating capacities of RBPH-B, RBPH-C, and their protein fractions are shown in Table 2. In contrast to the ABTS^+^ and DPPH results, ascorbic acid showed the lowest metal chelating ability. Notably, the protein fractions with smaller molecular sizes demonstrated higher chelating activities. The highest activity was observed for RBPH-C-F4 (1.35 mmol EDTA/g), followed by RBPH-C-F3 and RBPH-B-F4. Both hydrolysates (RBPH-B and RBPH-C) showed the lowest chelating activity compared to their fractions, indicating that the enhanced metal-binding ability is associated with the generation of smaller peptides. RBPH-C-F4 consistently demonstrated strong chelating activity across all tests, which may be attributed to its specific amino acid composition. Peptides rich in amino acids such as histidine, lysine, arginine, glutamic acid, and aspartic acid are known to possess strong metal-binding capacities due to the presence of side chains capable of coordinating metal ions [28,38]. This finding aligns with previous studies showing that smaller peptides derived from sesame protein hydrolysates [5] and tilapia protein [39] exhibited superior metal chelating activities. However, Phongthai et al. [3] reported that smaller rice bran peptides were less effective at binding iron ions, suggesting that metal-chelating ability depends not only on peptide size but also on other factors such as the type of protease used, hydrolysis conditions, and the specific protein source.

#### 3.3.4. Lipid Peroxidation Inhibition Activity

The inhibition of linoleic acid peroxidation by the peptides is shown in Table 2. While ascorbic acid exhibited limited inhibition in this assay, smaller peptides showed significantly higher activity. The extent of inhibition decreased in the following order: RBPH-B-F4 (77.08%) > RBPH-B-F3 (54.22%) > RBPH-B-F2 (49.85%) and RBPH-B-F1 (47.84%) > RBPH-B (35.99%); likewise, RBPH-C-F4 (90.62%) > RBPH-C-F3 (70.60%) > RBPH-C-F2 (57.57%) > RBPH-C (46.57%) > RBPH-C-F1 (42.50%). These results highlight the strong lipid antioxidant potential of low-molecular-weight protein fractions, particularly RBPH-C-F4. Their high efficacy may stem from their enhanced water solubility, as well as the presence of more exposed reactive amino acid side chains that can inhibit lipid oxidation. Previous studies have similarly shown the effectiveness of <1 kDa peptides from sesame [5], mushroom [30], and kidney bean [28] in suppressing lipid peroxidation.

In summary, enzymatic hydrolysis using *Bacillus licheniformis* and α-chymotrypsin, followed by ultrafiltration, significantly improved the antioxidant potential of rice bran peptides. Smaller peptides fractions (<1 kDa) consistently exhibited the highest ABTS and DPPH radical scavenging activities, metal chelating capacity, and lipid peroxidation properties. While the present study provides insight into how enzyme specificity and peptide size distribution influence antioxidant behavior, direct identification of bioactive peptide sequences was not performed. LC-MS/MS-based peptides profiling will be essential in future studies to establish definitive sequence–structure–activity relationships. These findings underscore the value of tailored enzymatic processing and membrane separation to generate multifunctional antioxidant peptides for use in food and nutraceutical applications.

## 4. Conclusions

This study demonstrated that enzymatic hydrolysis of rice bran protein using *Bacillus licheniformis* and α-chymotrypsin, followed by ultrafiltration significantly, altered polypeptide characteristics, including molecular weight distribution, surface hydrophobicity, secondary structure, and phenolic content. The peptides produced, particularly the smallest ones (<1 kDa), had enhanced antioxidant properties. Among all samples, the α-chymotrypsin-derived fraction RBPH-C-F4 exhibited the highest ABTS^+^, metal chelating, and linoleic peroxidation inhibition activities. These results highlight the potential of rice bran-derived peptides as plant-derived antioxidant ingredients for functional food and nutraceutical applications. Further investigations incorporating peptide profiling, process yield assessment, and validation in model food matrices or emulsions are required to confirm the functional relevance of these peptides under practical application conditions.

## Figures and Tables

**Table 1:** Color characteristics of rice bran protein (RBP), rice bran protein hydrolysate produced using *Bacillus licheniformis* (RBPH-B), and α-chymotrypsin (RBPH-C), and their protein fractions (F1–F4).

Samples	L*	a*	b*	ΔE*
RBP	54.14 ± 0.69 ^G^	4.40 ± 0.59 ^A^	13.03 ± 0.18 ^F^	^-^
RBPH-B	69.12 ± 0.33 ^bF^	2.61 ± 0.16 ^aB^	15.20 ± 0.58 ^dE^	15.25 ± 0.33 ^dG^
RBPH-B-F1	70.23 ± 1.85 ^bEF^	2.56 ± 0.20 ^aB^	16.69 ± 0.01 ^cD^	16.61 ± 1.80 ^cdF^
RBPH-B-F2	70.78 ± 0.44 ^bE^	2.53 ± 0.19 ^aB^	17.52 ± 0.30 ^cC^	17.34 ± 0.36 ^cF^
RBPH-B-F3	73.58 ± 0.42 ^aD^	2.01 ± 0.19 ^bC^	18.39 ± 0.54 ^bB^	20.31 ± 0.52 ^bE^
RBPH-B-F4	75.04 ± 0.42 ^aC^	1.65 ± 0.32 ^bC^	19.61 ± 0.65 ^aA^	22.09 ± 0.25 ^aC^
RBPH-C	75.33 ± 0.80 ^cC^	0.16 ± 0.05 ^aD^	13.11 ± 0.18 ^cF^	21.61 ± 0.78 ^cCD^
RBPH-C-F1	74.39 ± 0.02 ^dCD^	0.16 ± 0.05 ^aD^	13.14 ± 0.15 ^cF^	20.69 ± 0.02 ^dDE^
RBPH-C-F2	75.36 ± 0.02 ^cC^	0.20 ± 0.01 ^aD^	13.36 ± 0.08 ^bF^	21.63 ± 0.02 ^cCD^
RBPH-C-F3	81.29 ± 0.07 ^bB^	−0.60 ± 0.16 ^cE^	14.95 ± 0.04 ^aE^	27.67 ± 0.10 ^bB^
RBPH-C-F4	82.97 ± 0.00 ^aA^	−0.33 ± 0.03 ^bE^	14.99 ± 0.01 ^aE^	29.28 ± 0.00 ^aA^

Note: Values are presented as mean (n = 9) ± standard deviation (SD). Different lowercase letters within the same enzymatic treatment indicate significant difference (*p* < 0.05) in the same column. Different uppercase letters indicate significant difference (*p*< 0.05) across all samples in the same columns. Protein fractions: F1 (>100 kDa), F2 (10–100 kDa), F3 (1–10 kDa), and F4 (<1 kDa).

**Table 2:** Antioxidant activities of rice bran protein hydrolysates produced by *Bacillus licheniformis* (RBPH-B), and produced by α-chymotrypsin, and their protein fractions (F1–F4).

Samples	EC_50_		Metal Chelating Activity (mmol EDTA/g Sample)	Inhibition of Linoleic Acid Peroxidation (%)
	ABTS^+^ (mg/mL)	DPPH (µg/mL)		
Ascorbic acid	0.19 ± 0.00 ^I^	2.42 ± 0.15 ^G^	0.04 ± 0.00 ^G^	18.99 ± 1.21 ^J^
RBPH-B	3.01 ± 0.13 ^aA^	908.60 ± 20.58 ^aA^	0.77 ± 0.02 ^cF^	35.99 ± 1.71 ^dI^
RBPH-B-F1	2.58 ± 0.11 ^bB^	475.97 ± 28.20 ^bB^	0.79 ± 0.01 ^cEF^	47.84 ± 0.17 ^cG^
RBPH-B-F2	2.30 ± 0.05 ^cC^	210.50 ± 10.38 ^cF^	0.84 ± 0.01 ^bDE^	49.85 ± 1.02 ^cF^
RBPH-B-F3	1.99 ± 0.04 ^dD^	199.96 ± 6.43 ^cF^	0.89 ± 0.02 ^aC^	54.22 ± 0.84 ^bE^
RBPH-B-F4	1.84 ± 0.01 ^dE^	202.44 ± 9.07 ^cF^	0.92 ± 0.04 ^aC^	77.08 ± 1.61 ^aB^
RBPH-C	1.99 ± 0.03 ^aD^	399.80 ± 4.67 ^aC^	0.88 ± 0.02 ^cCD^	46.57 ± 0.13 ^dG^
RBPH-C-F1	1.98 ± 0.02 ^aD^	329.50 ± 7.67 ^bD^	0.93 ± 0.07 ^cC^	42.50 ± 0.49 ^eH^
RBPH-C-F2	1.60 ± 0.05 ^bF^	300.80 ± 7.10 ^cE^	1.02 ± 0.02 ^bB^	57.57 ± 1.41 ^cD^
RBPH-C-F3	1.37 ± 0.03 ^cG^	280.83 ± 4.04 ^dE^	1.06 ± 0.01 ^bB^	70.60 ± 0.55 ^bC^
RBPH-C-F4	0.94 ± 0.04 ^dH^	210.53 ± 2.02 ^eF^	1.35 ± 0.05 ^aA^	90.62 ± 0.15 ^aA^

Note: The results are presented as the mean (n = 9) ± SD. Different lowercase letters indicate significantly different (*p* < 0.05) within the same columns of the same protease hydrolysis, while different uppercase letters indicate significantly different (*p* < 0.05) within the same columns overall.

## Data Availability

The original contributions presented in the study are included in the article. Further inquiries can be directed to the corresponding author.
